# Neuropharmacology of Cannabinoids: A Comprehensive Review of Preclinical and Clinical Evidence for Hemp-Derived Extracts and Active Compounds

**DOI:** 10.3390/ph19081151

**Published:** 2026-07-24

**Authors:** Charles A. Odonkor, David A. Karpe, Muhammad Uzair Siddique, Alaa Abd-Elsayed

**Affiliations:** 1 Department of Orthopedics and Rehabilitation, Division of Physiatry, Interventional Pain Medicine, Yale New Haven Hospital and Yale University School of Medicine, New Haven, CT 06510, USA; 2 University of New England College of Osteopathic Medicine, Biddeford, ME 04005, USA; dkarpe@une.edu; 3 Department of Orthopedics and Rehabilitation, Yale University School of Medicine, New Haven, CT 06510, USA; muhammaduzair.siddique@yale.edu; 4 Department of Anesthesiology, University of Wisconsin, Madison, WI 53792, USA; abdelsayed@wisc.edu

**Keywords:** cannabinoids, cannabidiol, tetrahydrocannabinol, endocannabinoid system, neuropharmacology, neuroprotection, epilepsy, multiple sclerosis, neuropathic pain, cannabis use disorder

## Abstract

*Cannabis sativa* contains more than 120 phytocannabinoids, with Δ9-tetrahydrocannabinol (THC) and cannabidiol (CBD) being the best characterized. This review synthesizes preclinical and clinical evidence on hemp-derived extracts, cannabinoids, and active compounds. THC primarily acts as a partial agonist at cannabinoid receptor type 1 (CB1) and type 2 (CB2), producing psychoactive, appetite-stimulating, antiemetic, and analgesic effects. CBD is non-intoxicating and has a multimodal profile involving CB1 negative allosteric modulation, CB2 inverse agonism or antagonism, inhibition of anandamide inactivation, and activity at 5-HT1A receptors, transient receptor potential channels, GPR55, and peroxisome proliferator-activated receptor gamma. Preclinical models of Parkinson’s disease, Alzheimer’s disease, Huntington’s disease, epilepsy, and pain support anti-inflammatory, antioxidant, anti-excitotoxic, and glial-modulating mechanisms, but clinical translation remains uneven. The strongest evidence supports FDA-approved cannabidiol for Lennox–Gastaut syndrome, Dravet syndrome, and tuberous sclerosis complex, and THC-based agents for refractory chemotherapy-induced nausea and vomiting and AIDS-related anorexia. Moderate-certainty evidence supports nabiximols for multiple sclerosis spasticity and small benefits in selected chronic neuropathic pain populations. Evidence remains insufficient or negative for acute pain, insomnia, most psychiatric disorders, and many promoted indications. Key risks include cannabis use disorder, cognitive and psychiatric effects, cardiovascular events, sedation, high-dose CBD hepatotoxicity, and drug interactions. Rigorous, long-term, product-standardized trials are needed.

## 1. Introduction

The medicinal use of *Cannabis sativa* spans more than four millennia, with documented applications in Chinese, Indian, Middle Eastern, and Greco-Roman pharmacopeias for pain, spasms, and convulsive disorders. Cannabis entered Western medicine in the mid-nineteenth century through the work of William O’Shaughnessy, was listed in the United States Pharmacopeia until 1942, and was subsequently displaced by regulatory restriction and the rise in synthetic analgesics and anticonvulsants. The isolation and structural elucidation of Δ9-tetrahydrocannabinol (THC) by Gaoni and Mechoulam in 1964, followed by the cloning of the cannabinoid receptors and the discovery of the endogenous cannabinoid (endocannabinoid) system in the early 1990s, transformed cannabis from a botanical remedy into a defined neuropharmacological target [1,2]. The original isolation of Δ9-THC, the cloning of the CB1 receptor, and the identification of anandamide are the foundational primary reports underpinning this field [3,4,5].

Three convergent developments have driven contemporary interest in cannabinoid therapeutics: the global expansion of medical and recreational cannabis access, the regulatory distinction between hemp-derived and marijuana-derived products, and the approval of purified, pharmaceutical-grade cannabinoid medicines. In the United States, the 2018 Agriculture Improvement Act (“Farm Bill”) removed hemp—defined as *Cannabis sativa* containing ≤0.3% Δ9-THC by dry weight—from the Controlled Substances Act, catalyzing a large market in CBD-containing consumer products. At the same time, more than three dozen U.S. states and numerous national jurisdictions have established medical cannabis programs operating outside the federal drug-approval framework, producing a wide and largely unstandardized array of products [1,6].

This regulatory heterogeneity is central to interpreting the evidence base. “Cannabis-based products” encompass at least three distinct categories with markedly different levels of supporting data: (i) FDA-approved, pharmaceutical-grade single-molecule or standardized-extract medicines (cannabidiol oral solution, dronabinol, nabilone, and—outside the United States—nabiximols); (ii) state-regulated medical cannabis products of variable and often inaccurately labeled composition; and (iii) widely marketed hemp-derived CBD wellness products with minimal regulatory oversight [1,6,7]. Conflating these categories is a recurrent source of confusion in both the lay and scientific literature.

The objective of this review is to provide a rigorous, mechanistically grounded, and clinically applicable synthesis of the neuropharmacology of phytocannabinoids. We integrate phytochemistry, molecular and systems neuropharmacology, preclinical neuroprotection and pain data, and the current clinical evidence base, organized by condition, with explicit attention to effect sizes, certainty of evidence, dosing, safety, and professional-society recommendations. We emphasize hemp- and cannabis-derived extracts and their active compounds rather than synthetic high-potency cannabinoid receptor agonists, which carry a distinct and substantially more dangerous toxicology profile.

Several reviews of cannabinoid pharmacology and therapeutics have been published; this synthesis is distinguished from prior works in four respects. First, rather than treating “cannabis” as a single entity, we explicitly separate the evidence for FDA-approved, pharmaceutical-grade cannabinoid medicines from that for state-regulated medical cannabis and minimally regulated consumer CBD products—a distinction frequently blurred in the existing literature. Second, we integrate phytochemistry, molecular and systems neuropharmacology, and preclinical neuroprotection data with a condition-by-condition appraisal of the clinical evidence, linking specific receptor-level mechanisms to clinical outcomes rather than reviewing mechanism or efficacy in isolation. Third, we report effect sizes and the certainty of evidence alongside professional-society and regulatory recommendations, yielding a clinically actionable rather than a purely descriptive account. Fourth, we focus on hemp- and cannabis-derived extracts and their active constituents while deliberately excluding synthetic high-potency receptor agonists, whose toxicology is distinct and substantially more dangerous. To our knowledge, few prior reviews combine this mechanistic depth, explicit grading of evidence, and regulatory framing within a single, up-to-date (through 2026) source. Specifically, and in contrast to earlier narrative reviews, we (i) grade the certainty of evidence condition-by-condition, (ii) keep preclinical and clinical evidence streams explicitly separate (summarized side-by-side in below), and (iii) treat the preclinical-to-clinical translational gap and pervasive product heterogeneity as primary interpretive lenses rather than closing caveats.

Because the discrepancy between preclinical promise and clinical performance recurs across every indication discussed below, we flag it at the outset. Preclinical findings—typically derived from acute lesion paradigms in young, genetically uniform rodents—should be read as hypothesis-generating for human disease, and we therefore report and interpret them separately from clinical data throughout.

## 2. Methods

### Search Strategy and Selection Criteria

For this comprehensive narrative review, we conducted a structured literature search to enhance transparency and reproducibility. We searched MEDLINE/PubMed, Embase, the Cochrane Central Register of Controlled Trials and Cochrane Database of Systematic Reviews, and Scopus from database inception to May 2026, supplemented by hand-searching of reference lists, regulatory sources (the U.S. FDA Orange Book and product labeling), and clinical-practice guidelines from professional societies. Search terms combined controlled vocabulary and free-text keywords for the exposure (“cannabinoid*”, “cannabidiol”, “CBD”, “tetrahydrocannabinol”, “THC”, “nabiximols”, “dronabinol”, “nabilone”, “*Cannabis sativa*”, “hemp”, “endocannabinoid”, “phytocannabinoid”) with terms for mechanism and indication (“neuroprotection”, “endocannabinoid system”, “epilepsy”, “multiple sclerosis”, “neuropathic pain”, “Parkinson”, “Alzheimer”, “anxiety”, “psychosis”, “nausea”, “safety”, “adverse effects”). The final search was executed on 15 May 2026. As a representative example, the MEDLINE (PubMed) strategy combined the exposure and outcome blocks as: (“cannabinoid*”[tiab] OR “cannabidiol”[tiab] OR “CBD”[tiab] OR “tetrahydrocannabinol”[tiab] OR “THC”[tiab] OR “nabiximols”[tiab] OR “dronabinol”[tiab] OR “nabilone”[tiab] OR “Cannabis sativa”[MeSH] OR “endocannabinoid*”[tiab] OR “phytocannabinoid*”[tiab]) AND (“neuroprotection”[tiab] OR “endocannabinoid system”[tiab] OR “epilepsy”[MeSH] OR “multiple sclerosis”[MeSH] OR “neuralgia”[MeSH] OR “Parkinson*”[tiab] OR “Alzheimer*”[tiab] OR “anxiety”[tiab] OR “psychosis”[tiab] OR “nausea”[tiab] OR “safety”[tiab] OR “adverse effect*”[tiab]), adapted to the syntax of each database.

Search results were screened by title and abstract, followed by full-text review of records considered directly relevant to cannabinoid neuropharmacology, therapeutic use, or safety. We included English-language preclinical studies, randomized controlled trials, systematic reviews and meta-analyses, umbrella reviews, regulatory documents, and evidence-based clinical-practice guidelines. We prioritized the highest available level of evidence and the most recent syntheses from 2018 to 2026, giving precedence to systematic reviews, meta-analyses, and guidelines over individual primary studies where these overlapped, while retaining seminal mechanistic and historical references regardless of date. We excluded non-peer-reviewed material, conference abstracts without full reports, case reports except where they represented the only available human data for an emerging indication, and studies of synthetic high-potency receptor agonists outside the review’s scope. Because this manuscript is a comprehensive narrative review rather than a registered systematic review or meta-analysis, no PRISMA flow diagram, formal risk-of-bias assessment, or quantitative pooling was performed. Records were screened independently by two reviewers (M.U.S. and D.A.K.), and disagreements at the full-text stage were resolved by a third author (C.A.O.) acting as arbiter. The search and hand-searching identified approximately 3200 records; after removal of duplicates, roughly 2650 were screened by title and abstract, of which about 320 full-text articles were assessed for eligibility, and 88 sources are cited in this review. Throughout, we specify study design (preclinical, randomized controlled trial [RCT], systematic review/meta-analysis, umbrella review, regulatory document, or clinical-practice guideline) and, where trials exist, the direction and certainty of the pooled estimate, rather than relying on non-specific phrasing such as “evidence supports.” To convey the balance and trajectory of the field: of the full-text records assessed, approximately three-fifths were basic or preclinical/mechanistic studies and roughly two-fifths were clinical (randomized trials, systematic reviews/meta-analyses, and guidelines). The annual volume of cannabinoid neuropharmacology publications has risen steeply since the 2018 U.S. approval of cannabidiol and the 2018 hemp legislation, with growth concentrated in epilepsy, chronic pain, and psychiatric indications; this is reflected in the predominance of post-2018 sources cited here. Of the 88 cited references, 25 are primary experimental or clinical studies, 18 are systematic reviews or meta-analyses, 11 are regulatory or clinical-practice-guideline documents, and 34 are narrative reviews.

## 3. Results and Discussion

### 3.1. Phytochemistry and Active Compounds

#### 3.1.1. Major Phytocannabinoids

Phytocannabinoids are terpenophenolic compounds—most bearing a 21-carbon skeleton in the pentyl series, as exemplified by THC, CBD, CBG, and CBC—derived from a common biosynthetic pathway. Geranyl pyrophosphate and olivetolic acid are condensed to form cannabigerolic acid (CBGA), the central precursor, which is then converted by three oxidocyclase enzymes—tetrahydrocannabinolic acid synthase (THCAS), cannabidiolic acid synthase (CBDAS), and cannabichromenic acid synthase (CBCAS)—into THCA, cannabidiolic acid (CBDA), and cannabichromenic acid (CBCA), respectively. The corresponding divarinic (C3 side-chain) precursor, cannabigerovarinic acid, yields the varin homologs such as tetrahydrocannabivarinic and cannabidivarinic acids [8,9].

In the living plant, cannabinoids accumulate predominantly as their carboxylic acid forms (THCA, CBDA, and CBGA), which are non-intoxicating. Non-enzymatic decarboxylation upon heating, combustion, or prolonged storage converts these acids to their neutral, pharmacologically active counterparts—THC, CBD, and cannabigerol (CBG)—a transformation of direct relevance to product formulation, dosing, and analytical quality control [8,10].

**Figure 1:**
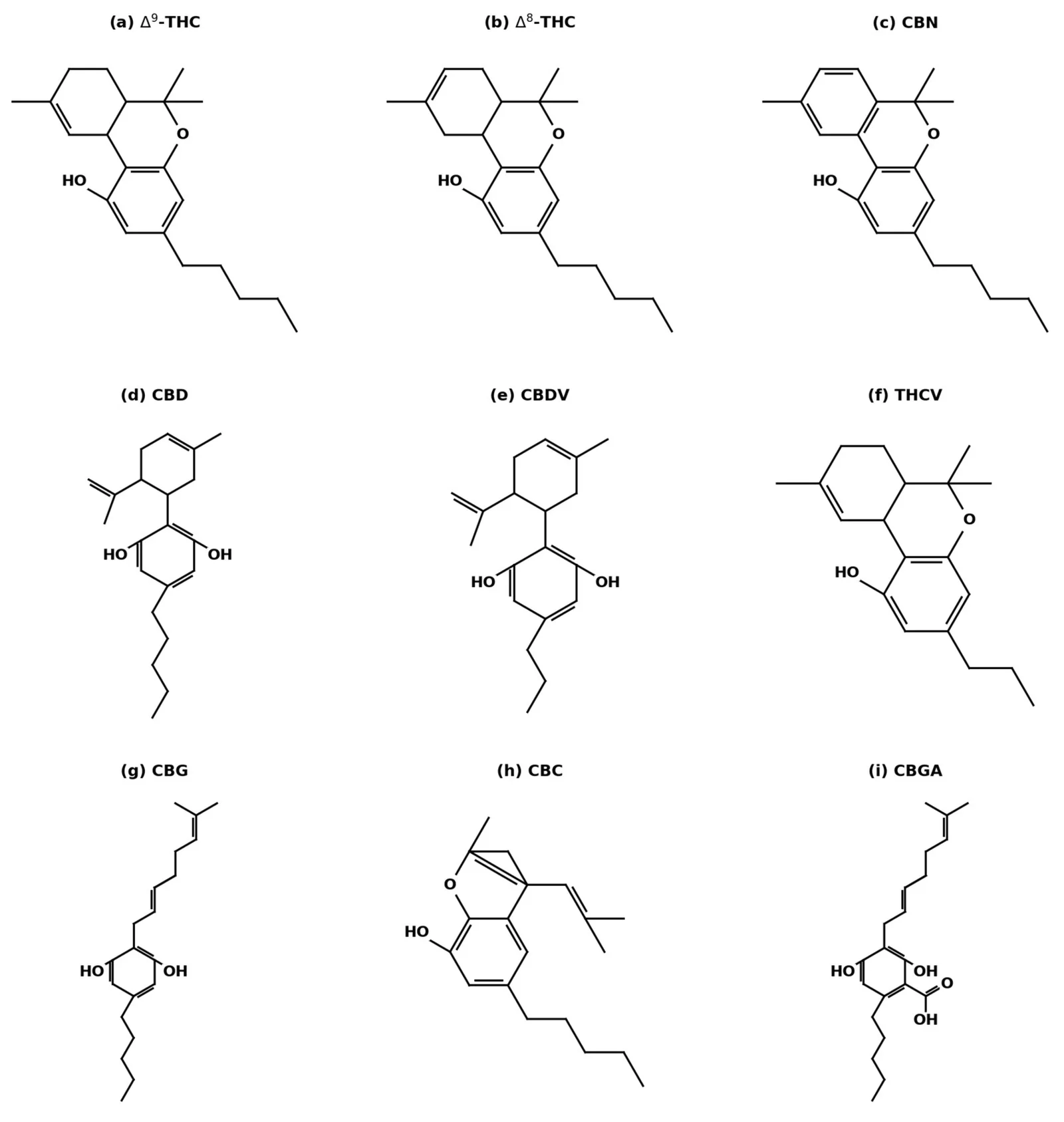
Chemical structures of representative phytocannabinoids. (**a**) Δ^9^-tetrahydrocannabinol (Δ^9^-THC); (**b**) Δ^8^-THC; (**c**) cannabinol (CBN); (**d**) cannabidiol (CBD); (**e**) cannabidivarin (CBDV); (**f**) tetrahydrocannabivarin (THCV); (**g**) cannabigerol (CBG); (**h**) cannabichromene (CBC); (**i**) cannabigerolic acid (CBGA), the common biosynthetic precursor. The varin homologues (CBDV, THCV) carry a C3 propyl side chain in place of the C5 pentyl chain; Δ^8^- and Δ^9^-THC are positional isomers differing in the location of the cyclohexene double bond.

Δ9-THC is the principal intoxicating constituent of cannabis and the molecule responsible for its characteristic subjective effects, appetite stimulation, antiemesis, and a substantial portion of its analgesic activity. CBD, the most abundant cannabinoid in hemp chemotypes, is non-intoxicating and accounts for much of the anticonvulsant and anxiolytic profile attributed to cannabis extracts. The relative proportions of THC and CBD define plant chemotypes and are the primary determinants of a product’s clinical and abuse-liability profile [1,2,11]. Additional structurally related compounds of pharmacological interest include CBG and cannabichromene (CBC), the propyl homologs cannabidivarin (CBDV) and tetrahydrocannabivarin (THCV), and Δ8-THC, a position isomer of Δ9-THC with reduced potency that is frequently synthesized from CBD for the consumer market [12,13,14,15]. The chemical structures of the major phytocannabinoids, together with representative minor cannabinoids and their acidic biosynthetic precursor, are shown in Figure 1.

#### 3.1.2. Minor Cannabinoids and Other Constituents

More than 120 phytocannabinoids have been identified across at least eleven chemical subclasses, although the vast majority occur at trace concentrations and have not been pharmacologically characterized [8,12]. Beyond the cannabinoids, the plant produces more than 150 terpenes and a range of flavonoids that contribute to its aroma, chemovar identity, and, potentially, its pharmacology. Monoterpenes such as β-myrcene, limonene, α- and β-pinene, and linalool, and the sesquiterpene β-caryophyllene—a dietary cannabinoid that is itself a selective CB2 agonist—have demonstrated anti-inflammatory, anxiolytic, and antinociceptive activities in preclinical models [16,17,18,19].

These observations underpin the “entourage effect” hypothesis, which posits that the therapeutic activity of whole-plant extracts reflects synergistic or modulatory interactions among cannabinoids, terpenes, and flavonoids rather than the action of any single molecule. Cannabis-derived terpenes, including α-humulene, geraniol, linalool, and β-pinene, have shown cannabimimetic activity in mice and have additive effects with cannabinoid receptor agonists, with some behaviors blocked by cannabinoid or adenosine receptor antagonists [17]. While mechanistically plausible and supported by selected preclinical data, the entourage effect remains incompletely validated in humans, and its invocation to justify unstandardized full-spectrum products should be interpreted cautiously [20,21].

### 3.2. Neuropharmacological Mechanisms

#### 3.2.1. The Endocannabinoid System

**Figure 2:**
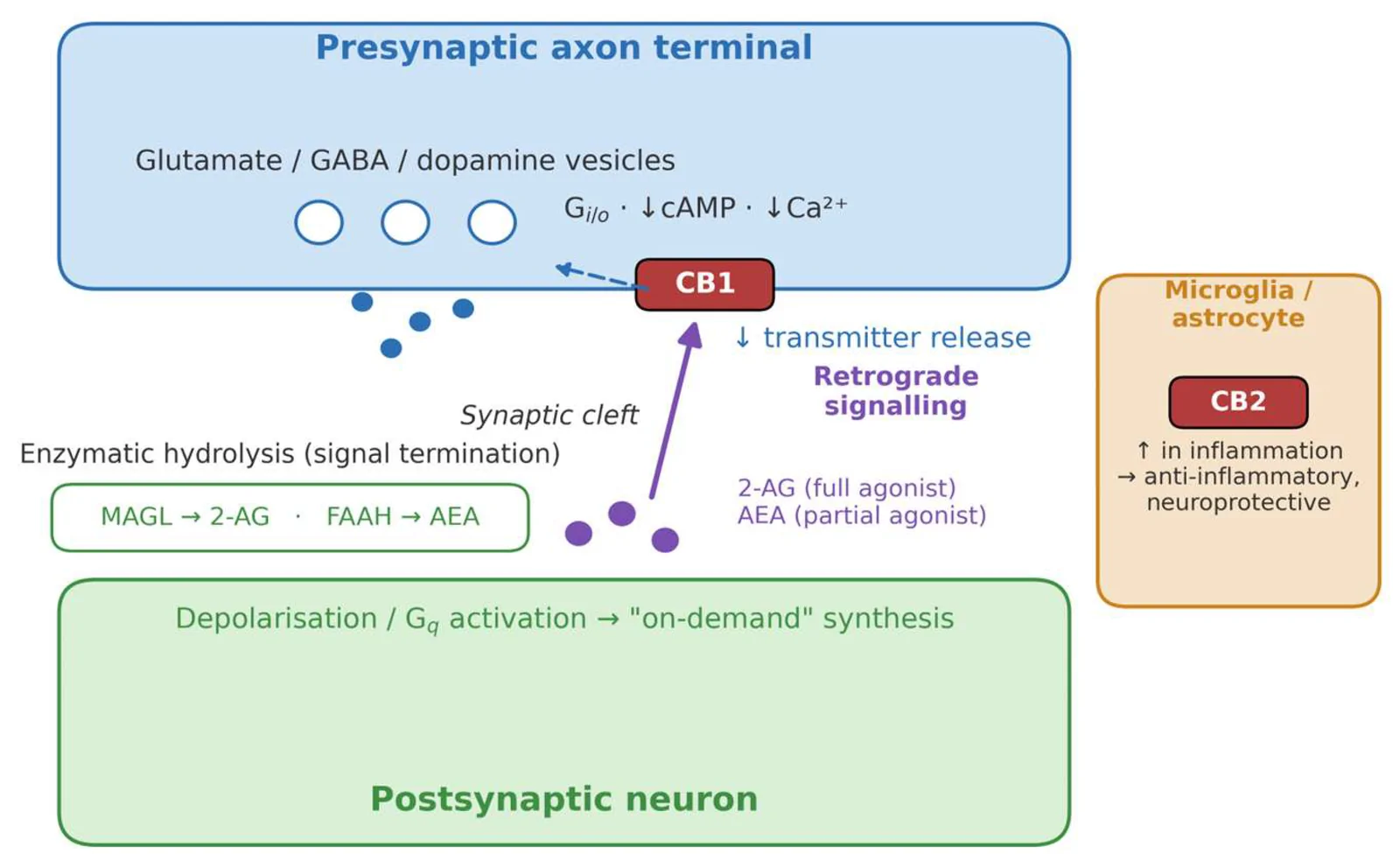
The endocannabinoid system: retrograde synaptic signaling and its principal targets. Postsynaptic depolarization triggers on-demand synthesis of the endocannabinoids 2-AG and anandamide (AEA), which act retrogradely at presynaptic CB1 receptors to suppress neurotransmitter release; signaling is terminated by MAGL and FAAH. CB2 receptors, upregulated on activated microglia and astrocytes during inflammation, mediate anti-inflammatory and neuroprotective effects. Arrows: → denotes the direction of signaling or enzymatic conversion; ↑ and ↓ denote increase and decrease, respectively. Colors denote cellular compartments and are schematic: blue, presynaptic axon terminal; green, postsynaptic neuron; orange, microglia/astrocyte; red, cannabinoid receptors (CB1, CB2).

The endocannabinoid system (ECS) is an evolutionarily conserved lipid-signaling network that maintains homeostasis across the nervous, immune, gastrointestinal, and endocrine systems. Its core components are two G protein-coupled cannabinoid receptors (CB1 and CB2), two principal endogenous ligands—N-arachidonoylethanolamine (anandamide, AEA) and 2-arachidonoylglycerol (2-AG)—and the enzymes that synthesize and degrade them [22,23]. The core architecture of the system and its retrograde mode of signaling are depicted in Figure 2.

CB1 is among the most abundant G protein-coupled receptors in the central nervous system, with dense expression in the cerebral cortex, hippocampus, amygdala, basal ganglia, substantia nigra, globus pallidus, and cerebellum, as well as along central and peripheral pain pathways. It is localized predominantly to presynaptic axon terminals, where its activation suppresses neurotransmitter release. CB2 is expressed at low levels in the healthy CNS. Still, it is markedly upregulated on activated microglia and infiltrating immune cells under inflammatory and neurodegenerative conditions, making it an attractive target for neuroprotection without psychoactivity [22,23,24].

Endocannabinoid signaling is principally retrograde: postsynaptic depolarization and Gq-coupled receptor activation trigger “on-demand” synthesis of 2-AG (and AEA), which travel backward across the synapse to activate presynaptic CB1 and restrain further transmitter release. 2-AG is a full agonist at both CB1 and CB2 and is present in the brain at concentrations roughly two orders of magnitude higher than AEA, which behaves as a high-affinity partial agonist. Signaling is terminated by enzymatic hydrolysis—fatty acid amide hydrolase (FAAH) degrades AEA, whereas monoacylglycerol lipase (MAGL) is responsible for the majority of 2-AG hydrolysis—generating arachidonic acid and feeding into eicosanoid pathways [22,23].

#### 3.2.2. Receptor Pharmacology of the Major Cannabinoids

THC and CBD, despite their close structural relationship, produce divergent neuropharmacological outcomes because they engage the ECS through fundamentally different mechanisms [2]. THC is a partial agonist at both CB1 and CB2. Its CB1 agonism in cortico-limbic and mesolimbic circuits accounts for euphoria, altered perception, appetite stimulation, and analgesia, but also for dysphoria, anxiety, cognitive impairment, and—at high doses or in vulnerable individuals—transient psychotic symptoms [1,2,11].

CBD has negligible direct agonist activity at orthosteric cannabinoid receptor sites. It functions as a negative allosteric modulator at CB1, attenuating the signaling of both THC and endogenous agonists, and as an inverse agonist/antagonist at CB2. CBD also inhibits the cellular reuptake and FAAH-mediated degradation of anandamide, thereby indirectly enhancing endocannabinoid tone. These properties appear to provide a mechanistic basis for CBD’s capacity to counteract several of the adverse effects of THC [2,25,26]. Preclinically, CBD has been reported to reverse THC-induced increases in dopaminergic neuronal firing and aberrant salience attribution by bidirectionally regulating ERK1/2 phosphorylation in the ventral hippocampus, and the two compounds exert dissociable effects on prefrontal executive function and affective regulation [27,28]. Table 1 contrasts the receptor-level pharmacology of THC and CBD directly.

#### 3.2.3. Non-Cannabinoid Receptor Targets

Much of CBD’s therapeutic profile is mediated by targets outside the classical cannabinoid receptors. CBD is an agonist at serotonin 5-HT1A receptors—a mechanism implicated in its anxiolytic, antidepressant, and neuroprotective actions—and modulates transient receptor potential (TRP) channels, including desensitization of TRPV1 and activation of TRPV2, TRPA1, and TRPM8, relevant to nociception and inflammation. CBD antagonizes the orphan receptor GPR55, potentiates glycine receptors, activates peroxisome proliferator-activated receptor gamma (PPAR-γ), and influences adenosine signaling through inhibition of adenosine reuptake [11,25,29]. These convergent, non-CB1 mechanisms—particularly TRPV1 modulation, GPR55 antagonism, and adenosine effects—are thought to underlie CBD’s anticonvulsant activity in treatment-resistant epilepsy [25,30].

#### 3.2.4. Neurotransmitter Modulation and Synaptic Plasticity

Through presynaptic CB1 receptors, cannabinoids modulate the release of glutamate, γ-aminobutyric acid (GABA), dopamine, acetylcholine, and norepinephrine, shaping both phasic neurotransmission and longer-term synaptic plasticity. Endocannabinoid-mediated short-term (depolarization-induced suppression of inhibition/excitation) and long-term depression are central mechanisms of activity-dependent plasticity in the hippocampus, cerebellum, and basal ganglia. By restraining excessive glutamatergic transmission, cannabinoid signaling can limit excitotoxicity. At the same time, its modulation of mesolimbic dopamine release contributes both to the rewarding properties of THC and to its potential to disrupt salience processing. Disruption of this finely tuned system, particularly during the protracted neurodevelopmental window of adolescence, provides a mechanistic rationale for the cognitive and psychiatric vulnerabilities associated with heavy cannabis exposure [2,31,32]. Table 2 summarizes the major phytocannabinoids, their principal molecular targets, key effects, and clinical or abuse profiles. Table 1 directly contrasts the neuropharmacology of THC and CBD.

**Table 1:** Contrasting neuropharmacology of Δ9-THC and CBD.

Property	Δ9-THC	CBD
CB1 receptor	Partial agonist (orthosteric)	Negative allosteric modulator; negligible orthosteric agonism
CB2 receptor	Partial agonist	Inverse agonist/antagonist
Endocannabinoid tone	Direct receptor activation	Increases anandamide via inhibition of reuptake and FAAH
Principal non-CB targets	Limited	5-HT1A agonism; TRPV1 desensitisation; GPR55 antagonism; PPAR-γ activation
Psychoactivity	Intoxicating (dose-dependent)	Non-intoxicating
Principal desired effects	Analgesia; antiemesis; appetite stimulation	Anticonvulsant; anxiolytic; anti-inflammatory
Principal adverse effects	Anxiety; cognitive impairment; psychotomimesis; abuse liability	Diarrhoea; somnolence; dose-dependent transaminase elevation
Representative approved product	Dronabinol; nabilone	Cannabidiol oral solution (Epidiolex)

CB, cannabinoid receptor; AEA, anandamide; FAAH, fatty acid amide hydrolase; 5-HT1A, serotonin 1A receptor; TRPV1, transient receptor potential vanilloid 1; PPAR-γ, peroxisome proliferator-activated receptor gamma. Directly contrasts THC and CBD (cf. Section 3.2); synthesised from Refs [1,2,11,25,26,33].

**Table 2:** Comparative receptor pharmacology and clinical profile of the major phytocannabinoids.

Compound	Principal Molecular Targets	Key Effects	Clinical/Abuse Profile	Level of Clinical Evidence
Δ9-THC	CB1 and CB2 partial agonist	Euphoria; analgesia; appetite stimulation; antiemesis; anxiety; cognitive impairment; psychotomimesis	Intoxicating; abuse liability; FDA-approved as dronabinol/nabilone	High (FDA-approved; RCTs for CINV and AIDS anorexia)
CBD	CB1 negative allosteric modulator; CB2 inverse agonist; 5-HT1A agonist; TRPV1/GPR55/PPAR-γ; AEA reuptake/FAAH inhibition	Anticonvulsant; anxiolytic; antipsychotic; anti-inflammatory; counteracts THC effects	Non-intoxicating; low abuse liability; FDA-approved (Epidiolex); hepatotoxicity at high dose	High (pivotal RCTs; FDA-approved for epilepsy)
CBG	CB1/CB2 partial agonist; 5-HT1A; PPAR-γ; α2-adrenergic	Neuroprotection; anti-inflammatory (preclinical)	Non-intoxicating; investigational	Preclinical only
CBC	TRPA1 agonist; AEA uptake inhibition; weak CB2	Antinociceptive; anti-seizure; anti-inflammatory (preclinical)	Non-intoxicating; investigational	Preclinical only
CBDV	TRP channels; 5-HT1A	Anticonvulsant; ASD-related behaviors (preclinical/early clinical)	Non-intoxicating; investigational	Preclinical; early-phase clinical
THCV	CB1 antagonist (low dose)/agonist (high dose); CB2	Anticonvulsant; possible metabolic and motor effects (preclinical)	Dose-dependent intoxication; investigational	Preclinical only

CB, cannabinoid receptor; 5-HT1A, serotonin 1A receptor; TRP, transient receptor potential; PPAR-γ, peroxisome proliferator-activated receptor gamma; AEA, anandamide; FAAH, fatty acid amide hydrolase; ASD, autism spectrum disorder. Synthesized from Refs [1,2,11,12,14,25].

### 3.3. Preclinical Evidence

#### 3.3.1. Animal Models and Experimental Paradigms

Preclinical cannabinoid neuroscience relies on a well-established repertoire of rodent and, less commonly, non-human primate (marmoset) models. Parkinsonian phenotypes are induced by the catecholaminergic neurotoxins 6-hydroxydopamine (6-OHDA, typically unilateral intrastriatal or medial forebrain bundle injection in rats) and 1-methyl-4-phenyl-1,2,3,6-tetrahydropyridine (MPTP, systemic in mice and primates), as well as by the mitochondrial complex I inhibitor rotenone, which reproduces oxidative stress and α-synuclein pathology. Neuroinflammation is modeled with lipopolysaccharide (LPS), Alzheimer’s pathology with transgenic amyloid/tau lines and amyloid-β infusion, and Huntington’s disease with the excitotoxins quinolinic and 3-nitropropionic acid. Seizure susceptibility is assessed with maximal electroshock, pentylenetetrazole, and genetic models, and outcomes are quantified with motor (rotarod, cylinder, open field), cognitive (novel object recognition, Morris water maze), and neurochemical assays [30,34,35]. These paradigms model discrete facets of human disease and, being largely acute, may overstate effects relative to chronic human neurodegeneration—a caveat that applies to all preclinical findings summarized below.

#### 3.3.2. Neuroprotective Effects in Models of Neurodegeneration

In Parkinson’s disease models, phytocannabinoids—principally CBD, but also THCV, CBG, and Δ9-THC—have repeatedly been reported to show dopaminergic neuroprotection and improvement of motor deficits. A 2024 systematic review of phytocannabinoids in animal models of Parkinson’s disease concluded that cannabinoid treatment generally preserved nigrostriatal integrity and motor function across 6-OHDA and MPTP paradigms [34]. CBD has been reported to reduce striatal terminal degeneration and substantia nigra cell loss in 6-OHDA-lesioned rats, accompanied by upregulation of antioxidant defenses such as copper/zinc superoxide dismutase [36]. CB2 receptor activation attenuates MPTP-induced microglial activation, whereas genetic deletion of CB2 exacerbates toxicity, and GPR55 signaling has been implicated in preventing dopaminergic cell death—illustrating the multiplicity of receptor pathways involved [36,37]. Quantitatively, in 6-OHDA-lesioned rats, CBD given immediately after the lesion recovered the depletion of dopamine and tyrosine-hydroxylase-positive neurons toward control values, an effect accompanied by increased Cu, Zn-superoxide dismutase mRNA expression [36]. In dopaminergic cell models, CBD pretreatment (5–10 µM) raised cell viability from approximately 48% to 82% of control and reduced apoptosis from roughly 37% to 13%, while significantly lowering malondialdehyde and reactive oxygen species and increasing superoxide dismutase and glutathione activity [38].

In Alzheimer’s disease models, cannabinoids reduce amyloid-β-associated neuroinflammation and oxidative injury, modulate tau hyperphosphorylation, and improve cognition: CBD reverses cognitive deficits in transgenic and amyloid-infusion rodents, low-dose THC improves cognition in aged mice, and combined CBD: THC regimens may outperform single agents [37,39]. Huntington’s disease models show neuroprotection with CBD, THCV, and CBG-derived compounds, and amyotrophic lateral sclerosis and traumatic brain injury/stroke models demonstrate prolonged survival, reduced lesion volume, and improved functional recovery, particularly with CB2-biased and antioxidant-acting cannabinoids [12,37,40,41].

#### 3.3.3. Mechanisms of Neuroprotection

Across these models, neuroprotection converges on several overlapping mechanisms. Anti-inflammatory effects appear to be largely mediated through CB2 receptors on microglia and astrocytes, shifting glia from a pro-inflammatory to a reparative phenotype and reducing the release of tumor necrosis factor-α, interleukins, and nitric oxide. Antioxidant effects arise from both receptor-independent redox chemistry of the cannabinoid phenolic structure and the upregulation of endogenous antioxidant systems. Cannabinoids limit glutamatergic excitotoxicity by restraining presynaptic glutamate release and modulating NMDA-receptor-mediated calcium influx, engage pro-survival signaling (PI3K/Akt, PPAR-γ), and can stabilize blood–brain barrier integrity under ischemic and inflammatory stress [24,35,37,41]. CBG and its synthetic derivatives act predominantly via PPAR-γ, and CBG’s protection against rotenone toxicity involves 5-HT1A signaling—a pathway distinct from that of CBD [12,29].

#### 3.3.4. Pain Modulation in Animal Models

Cannabinoids are antinociceptive across inflammatory (formalin, carrageenan, and complete Freund’s adjuvant) and neuropathic (chronic constriction injury, spinal nerve ligation, and chemotherapy-induced) pain models. Antinociception is mediated by CB1 receptors at supraspinal, spinal, and peripheral sites and by CB2 receptors on immune cells, with additional contributions from TRPV1 and glycine receptors. Several minor cannabinoids, including CBC and CBG, are cannabimimetic and antinociceptive in mouse models of chronic neuropathic pain, and dose–response relationships frequently follow biphasic or bell-shaped curves, with intermediate doses producing maximal analgesia—an important consideration for translation to human dosing [19,24,42].

### 3.4. Clinical Evidence by Condition

#### 3.4.1. FDA-Approved Indications

##### Treatment-Resistant Epilepsy

The strongest evidence base for any hemp-derived compound is for purified cannabidiol oral solution (Epidiolex) in treatment-resistant developmental and epileptic encephalopathies. Randomized, placebo-controlled trials established efficacy in Dravet syndrome [43,44] and Lennox–Gastaut syndrome [45], leading to FDA approval in 2018; approval was extended to seizures associated with tuberous sclerosis complex in 2020 on the basis of a further placebo-controlled RCT [46]. CBD is approved for patients aged ≥1 year [30,33]. Across pivotal trials and meta-analyses, adjunctive CBD produces a clinically meaningful reduction in seizure frequency, with a standardized mean difference of approximately −0.5 [1,30]. Antiseizure activity is independent of CB1 agonism and is attributed to TRPV1 desensitization, GPR55 antagonism, and modulation of adenosine and intracellular calcium signaling [25,30]. The principal adverse effects are somnolence, decreased appetite, diarrhea, and dose-dependent elevations in hepatic transaminases—the last occurring most often in patients co-treated with valproate and contributing to a pharmacokinetic interaction with clobazam (see Section 3.6.4) [33,47]. Quantitatively, in the pivotal Dravet syndrome trial, adjunctive CBD reduced monthly convulsive-seizure frequency by a median of 39% versus 13% with placebo, with 43% versus 27% of patients achieving at least a 50% reduction; in Lennox–Gastaut syndrome, CBD reduced monthly drop-seizure frequency by approximately 37–44% versus 17–22% with placebo [30,33]. At the level of disease pathophysiology, these targets converge on the core defect of these encephalopathies—pathological neuronal hyperexcitability and hypersynchronization. In Dravet syndrome, loss-of-function in the SCN1A-encoded Nav1.1 channel preferentially impairs GABAergic interneuron firing and disinhibits cortical networks; CBD counteracts this hyperexcitable state through mechanisms downstream of the primary channelopathy—desensitizing TRPV1, antagonizing GPR55 (which otherwise promotes presynaptic glutamate release), and augmenting adenosine-mediated inhibition and intracellular calcium buffering—thereby restraining the excitatory–inhibitory imbalance rather than correcting the underlying mutation. This distinction helps explain why CBD is effective across genetically heterogeneous encephalopathies: it acts on the shared network-level consequence (hyperexcitability) rather than on a single molecular cause [25,30].

##### Chemotherapy-Induced Nausea and Vomiting

The synthetic THC analogs dronabinol and nabilone were approved by the FDA in 1985 for chemotherapy-induced nausea and vomiting (CINV) refractory to conventional antiemetics. Most supporting trials predate modern 5-HT3- and NK1-receptor-antagonist regimens, limiting their contemporary relevance, and effect sizes for the cannabinoids are modest (standardized mean difference approximately −0.2 to −0.3) [1,48]. More recent randomized data have re-examined standardized oral THC: CBD extracts: a phase II crossover trial and a subsequent phase II/III trial demonstrated meaningful reductions in refractory and breakthrough CINV when added to guideline antiemetics, albeit with increased sedation and dizziness [49,50]. The 2024 American Society of Clinical Oncology (ASCO) guideline conditionally recommends dronabinol or nabilone as add-on therapy for refractory CINV, and current National Comprehensive Cancer Network (NCCN) antiemesis guidance lists cannabinoids among breakthrough options, while emphasizing that they are not first-line agents [48,51]. Mechanistically, the antiemetic effect maps onto the emetic circuitry: CB1 receptors are densely expressed in the dorsal vagal complex—the nucleus tractus solitarius and area postrema—where THC-mediated CB1 activation suppresses release of emetogenic neurotransmitters and dampens signaling upstream of the 5-HT3 and NK1 pathways that conventional antiemetics block distally. By reducing the afferent emetic drive itself rather than blocking a single downstream receptor, cannabinoids retain utility specifically in the refractory setting, where 5-HT3/NK1 antagonism has proven insufficient [1,48].

##### HIV/AIDS-Related Anorexia

Dronabinol is FDA-approved for anorexia associated with weight loss in patients with AIDS, based on trials demonstrating improved appetite and stabilization of body weight; the typical starting dose is 2.5 mg twice daily before meals. The magnitude of effect on body weight is moderate (standardized mean difference ≈ 0.5). Still, the overall certainty of evidence is very low, the supporting studies are dated, and contemporary antiretroviral therapy has substantially reduced the prevalence of AIDS wasting [1,52].

#### 3.4.2. Chronic Pain Conditions

##### Neuropathic Pain

Chronic neuropathic pain is the non-approved indication with the most robust supporting data. Systematic reviews and meta-analyses, including the BMJ rapid-recommendation review and serial Annals of Internal Medicine syntheses, indicate that cannabis-based medicines—most consistently the THC: CBD oromucosal spray nabiximols—produce a small improvement in pain, on the order of 0.5–1.0 points on a 0–10 scale, corresponding to a small, standardized effect [53,54,55]. A 2026 Cochrane review of cannabis-based medicines for chronic neuropathic pain reaffirmed small benefits accompanied by increased adverse events and dropout [56]. Among higher-THC products, nabilone has shown a moderate analgesic effect, whereas oral dronabinol has not consistently shown a separation from placebo [1,55]. The American College of Physicians (2025) issued a conditional recommendation that cannabis or cannabinoids may be considered for patients with painful neuropathy whose pain is inadequately controlled by first-line therapy, noting that benefits are small and that harms may outweigh them for many patients [57].

##### Cancer-Related Pain

Evidence for cannabinoids in cancer pain is weaker and largely negative for the primary outcome. Randomized trials of nabiximols added to opioids in advanced-cancer pain did not consistently meet their primary endpoints, and meta-analyses find no reliable analgesic benefit over placebo. The 2024 ASCO guideline, therefore, does not recommend cannabinoids for the treatment of cancer pain, while acknowledging that some patients may use them for symptom palliation and quality of life [48,53].

##### Other Chronic Pain Conditions

For fibromyalgia, osteoarthritis, and non-specific musculoskeletal pain, controlled evidence is sparse, heterogeneous, and generally insufficient to support routine use. The clearest exception within the broader pain domain is multiple sclerosis-related spasticity and associated pain, as discussed below. Observational and small randomized data in fibromyalgia suggest possible improvements in sleep and pain for some patients, but study quality is low, and the risk of bias is high [19,58,59].

#### 3.4.3. Neurological Disorders

##### Multiple Sclerosis

Nabiximols (2.7 mg THC + 2.5 mg CBD per oromucosal spray; marketed as Sativex outside the United States) is approved in numerous countries for moderate-to-severe multiple sclerosis (MS) spasticity inadequately controlled by first-line agents. A 2026 meta-analysis involving 8780 adults reported significant reductions in patient-reported spasticity (standardized mean difference ≈ −1.4) and pain [60]. The Cochrane review judged the evidence for nabiximol as an add-on therapy to be of moderate certainty for spasticity and pain, with smaller and less certain effects on bladder dysfunction and sleep. It noted that objective (clinician-rated) measures show smaller effects than patient-reported ones [61]. The National Institute for Health and Care Excellence (NICE) recommends a four-week therapeutic trial with continuation only if at least a 20% improvement in spasticity-related symptoms is achieved. Real-world cohorts report continuation rates above 90% at 12 weeks with an average of approximately 6 sprays per day, though long-term cognitive and psychiatric safety data remain limited [62,63].

##### Parkinson’s Disease

Despite compelling preclinical neuroprotection, clinical evidence in Parkinson’s disease is limited and inconsistent. Small trials and observational studies suggest possible benefit for non-motor symptoms—pain, sleep, and anxiety—and for levodopa-induced dyskinesia, but randomized data are insufficient to support cannabinoids for motor symptoms or disease modification, and orthostatic hypotension and cognitive effects are concerns in this older population [34,64].

##### Neurodegenerative Diseases

In Alzheimer’s and Huntington’s diseases, the gap between preclinical promise and clinical reality is wide. Small clinical trials of cannabinoids for agitation in dementia and for chorea and motor function in Huntington’s disease have generally failed to demonstrate significant benefit on primary outcomes, and optimal compounds, doses, and cannabinoid ratios remain undefined. The translational shortfall reflects differences in disease stage at treatment, dosing, blood–brain barrier penetration, and outcome selection between rodent models and human trials [31,37,39].

#### 3.4.4. Psychiatric and Behavioral Disorders

##### Anxiety Disorders

CBD has shown anxiolytic activity in experimental-anxiety paradigms (simulated public speaking) and in small, randomized trials of social anxiety and generalized anxiety disorder, including a placebo-controlled simulated-public-speaking RCT [65]. A meta-analysis of 316 patients reported a large anxiolytic effect (Hedges’ g ≈ −0.9), but the evidence derives from small, short trials and is insufficient for a clinical recommendation [66,67]. CBD’s dose–response for anxiety is characteristically inverted-U-shaped, with intermediate oral doses (≈300–600 mg) effective and lower or higher doses ineffective [68]. THC, by contrast, is frequently anxiogenic, especially at higher doses and in inexperienced users, and chronic high-potency use is associated with increased anxiety [1,69].

##### Post-Traumatic Stress Disorder (PTSD)

Mechanistic interest in the ECS for fear extinction has not translated into convincing clinical efficacy. Randomized evidence for cannabis or cannabinoids in PTSD is limited and largely negative or inconclusive, and the U.S. Department of Veterans Affairs/Department of Defense (VA/DoD) guideline recommends against cannabis for PTSD, citing insufficient evidence of benefit and potential for harm [70,71,72].

##### Autism Spectrum Disorder and Tourette Syndrome

Emerging but preliminary evidence suggests CBD-enriched extracts may improve irritability, social deficits, and disruptive behavior in some children with autism spectrum disorder, and THC-containing preparations have reduced tics in adults with Tourette syndrome in small trials. CBDV is in clinical development for autism. These signals require confirmation in adequately powered, controlled trials [13,73,74].

##### Sleep Disorders

Although improved sleep is among the most common reasons patients use cannabis, controlled evidence is weak. Where sleep improves, it is often secondary to relief of pain or spasticity rather than a primary hypnotic effect, and reported benefits must be weighed against tolerance, disruption of sleep architecture, and withdrawal-related insomnia with chronic use. Evidence is insufficient to recommend cannabinoids for primary insomnia [1,67].

##### Psychotic Disorders

Cannabinoids exert bidirectional effects on psychosis. THC—particularly high-potency products—acutely induces psychotomimetic symptoms and is associated dose-dependently with increased risk of psychotic disorders, especially with adolescent-onset and frequent use. CBD has shown a preliminary antipsychotic signal in early randomized trials [75,76], plausibly through mechanisms opposing THC, but the evidence is not yet sufficient to establish it as an antipsychotic. THC-containing products are contraindicated in individuals with, or at elevated risk of, psychotic illness [1,2,69,77].

#### 3.4.5. Conditions with Insufficient Evidence

For a range of additional promoted indications—acute pain, depression, attention-deficit/hyperactivity disorder, glaucoma, and inflammatory bowel disease—current controlled evidence is insufficient to support cannabinoid therapy, and in several cases (e.g., glaucoma, where the duration of intraocular-pressure lowering is impractically short), the evidence argues against use. For depression, preclinical signals exist [78], but controlled clinical evidence has not supported cannabinoid therapy, and heavy THC use may worsen mood symptoms [71,77]. Table 3 summarizes pharmaceutical-grade cannabinoid products, approved or best-evidence indications, representative dosing, and approximate effect sizes. For several of these indications, inadequate or highly variable pharmacokinetics—rather than a lack of target-level activity—may contribute to the weak or inconsistent clinical signal (Section 3.5.4).

### 3.5. Formulations, Pharmacokinetics, and Dosing

#### 3.5.1. Pharmaceutical Preparations and Quality Control

Cannabinoid products span a spectrum of regulatory rigor. At one end are pharmaceutical-grade medicines manufactured to defined specifications: the synthetic THC products dronabinol and nabilone, the purified plant-derived CBD oral solution Epidiolex, and the standardized whole-extract oromucosal spray nabiximols. At the other end are state-program medical cannabis and over-the-counter hemp CBD products, for which independent analyses have repeatedly documented substantial discrepancies between labeled and actual cannabinoid content, as well as contamination with pesticides, heavy metals, solvents, and—in the case of some hemp-derived Δ8-THC products—reaction byproducts of uncertain toxicology. This variability is a central limitation when extrapolating trial data to products available to patients [1,6,7].

#### 3.5.2. Routes of Administration and Pharmacokinetics

THC and CBD are highly lipophilic, and their disposition differs markedly by route of administration. Oral administration is subject to erratic absorption and extensive hepatic first-pass metabolism, yielding low and variable systemic bioavailability—approximately 4–12% for THC and 5–10% for CBD—with delayed, blunted peak concentrations (1–4 h). Oral THC is metabolized via CYP2C9, CYP2C19, and CYP3A4 to 11-hydroxy-THC, an active metabolite that is more potent than the parent and contributes to the pronounced psychoactivity of ingested products. Oromucosal and sublingual delivery partially bypasses first-pass metabolism, providing somewhat more predictable kinetics that underlie the design of nabiximols. Inhalation produces rapid onset and high peak concentrations. Still, it is not recommended for medical use because of dose imprecision and respiratory harms, and topical formulations achieve local but minimal systemic exposure [1,79].

#### 3.5.3. Dosing Strategies and Titration

All cannabinoid regimens follow a “start low, go slow” principle, individualized by indication. For CBD in epilepsy, the FDA label specifies weight-based dosing beginning at 2.5 mg/kg twice daily, increasing after one week to a maintenance dose of 5 mg/kg twice daily (10 mg/kg/day), with further weekly increments to a maximum of 10 mg/kg twice daily (20 mg/kg/day; 12.5 mg/kg twice daily for tuberous sclerosis complex). Substantial dose reductions are required in moderate-to-severe hepatic impairment; serum transaminases and bilirubin should be monitored at baseline and at 1, 3, and 6 months, and discontinuation should be gradual to avoid increased seizure frequency [33,47].

For investigational CBD in anxiety, the inverted-U dose–response means intermediate oral doses (≈300–600 mg) are most effective for acute anxiolysis, while chronic regimens of 150–300 mg/day have shown benefit in small trials; an open-label trial in treatment-resistant anxiety in young people (ages 12–25) titrated to 800 mg/day over 12 weeks achieved a 42.6% reduction in anxiety severity [66,80,81]. THC-containing products are initiated at 1–2.5 mg and titrated slowly; nabilone for CINV starts at 1 mg twice daily (label maximum 6 mg/day). Nabiximols is titrated by one spray per day to a typical maintenance of 6–8 sprays/day (≈16–22 mg THC and 15–20 mg CBD), with response assessed at four weeks [48,52,62].

For chronic pain, a modified-Delphi consensus and the BMJ rapid recommendation advise initiating a CBD-predominant oral extract (e.g., 5 mg twice daily, increasing by ≈10 mg every 2–3 days to ≈40 mg/day) before adding THC at 1–2.5 mg with slow titration, reserving higher THC doses for inadequate response and beginning any opioid taper only once functional improvement is evident [82,83]. Throughout, the central caveat from the ACP applies: for many patients with chronic noncancer pain, the small, expected benefit (≈0.5–1.0 points on a 0–10 scale) may not outweigh the harms [57]. Table 4 summarizes representative dosing and titration strategies by indication.

#### 3.5.4. Pharmacokinetic Determinants of Therapeutic Efficacy

A compound cannot engage its molecular targets unless it reaches them at adequate free concentrations, so pharmacokinetics—not pharmacodynamics alone—shapes therapeutic outcome, and pharmacokinetic limitations are a plausible contributor to the uneven clinical efficacy described above. This is especially relevant for CBD, whose broad target profile means that in vitro potency at any single site (5-HT1A, TRPV1, GPR55, PPAR-γ) may bear little relation to the free brain concentrations actually achieved in vivo. CBD oral bioavailability is low (≈5–10%) and highly variable, reflecting extensive first-pass metabolism to 7-hydroxy- and 7-carboxy-CBD and a pronounced food effect—a high-fat meal increases CBD exposure roughly four- to five-fold—so fasted-state dosing can yield sub-therapeutic concentrations. Because several CBD targets are engaged only at high (micromolar) concentrations that are difficult to attain in the brain at tolerated oral doses, robust in vitro and preclinical activity (for example in anxiety, psychosis, and neurodegeneration) may translate inconsistently to the clinic for pharmacokinetic rather than pharmacodynamic reasons. THC disposition is similarly route-dependent: oral THC is converted by CYP2C9/CYP2C19/CYP3A4 to the active, more potent, and more slowly cleared metabolite 11-hydroxy-THC, so the plasma parent concentration understates central effect, whereas inhalation produces high, transient peaks poorly matched to sustained target engagement. Sequestration in deep lipid compartments with slow redistribution, and CYP-mediated metabolic variability (Section 3.6.4), further decouple administered dose from target-site exposure. Accordingly, apparent pharmacodynamic “failures” in several indications may in part reflect inadequate absorption, distribution, or metabolic inactivation rather than a true absence of target-level activity, and future efficacy studies should report or model ADME parameters—including fed/fasted state and active-metabolite exposure—rather than administered dose alone [1,79].

### 3.6. Safety and Adverse Effects

#### 3.6.1. Short-Term Adverse Effects

The most frequent acute adverse effects of cannabinoids are dose-related and predominantly attributable to THC: dizziness, sedation and somnolence, dry mouth, and cognitive and psychomotor impairment affecting attention, memory, and reaction time. Gastrointestinal effects (nausea, vomiting, and diarrhea—the last prominent with high-dose CBD) and psychiatric effects ranging from euphoria to anxiety, dysphoria, and, at high doses, transient psychotic symptoms are common. Cardiovascular effects include tachycardia and orthostatic hypotension. In randomized trials, these events drive the elevated rates of withdrawal due to adverse effects seen with cannabinoids relative to placebo; most CNS reactions are dose-dependent and resolve within 1–3 days [1,52,57].

#### 3.6.2. Long-Term Safety Concerns

Cannabis use disorder (CUD) is the most consequential long-term risk. It is frequently underappreciated in medical contexts: a systematic review and meta-analysis estimated CUD prevalence at approximately 29% among people using medicinal cannabis, comparable to recreational users [84]. Heavy and adolescent-onset use is associated with persistent neurocognitive deficits and structural brain changes, and high-potency THC products are associated dose-dependently with increased risk of psychotic disorders [32,69,77]. The American Heart Association’s (AHA) 2022 scientific statement [32] and subsequent umbrella reviews link cannabis use to increased risks of myocardial infarction, stroke, and other cardiovascular events, and smoked cannabis carries respiratory harms analogous in some respects to tobacco. Tolerance and a defined withdrawal syndrome (irritability, sleep disturbance, and appetite change) develop with regular use [32,77].

Considered by domain, several long-term risks warrant individual emphasis. Cognitively, regular heavy use—particularly when initiated in adolescence—has been associated with persistent deficits in attention, memory, and executive function and with measurable structural and functional brain differences, though the degree to which these reverse with sustained abstinence remains debated. Psychiatrically, high-potency THC exposure is associated dose-dependently with incident psychotic disorders and with worsening anxiety and mood in susceptible individuals. Cardiovascularly, beyond acute tachycardia and orthostatic hypotension, cohort and umbrella-review data link regular use to elevated risks of myocardial infarction, stroke, and arrhythmia, and smoked preparations add respiratory harm. Finally, the dependence liability of THC-predominant products is substantial: tolerance and a defined withdrawal syndrome develop with regular use, and cannabis use disorder affects a clinically significant minority of medicinal users [32,69,77,84].

#### 3.6.3. Special Populations

Risk is concentrated in identifiable groups. Adolescents and young adults (≤25 years) are vulnerable to neurodevelopmental harm, cognitive impairment, and psychosis; a meta-analysis of 23 randomized trials (3612 children and adolescents receiving medical cannabinoids) found increased overall adverse events (RR 1.09), serious adverse events (RR 1.81), and withdrawals due to adverse events (RR 3.07), with somnolence and hepatotoxicity of particular concern [85]. Cannabis is contraindicated in pregnancy and lactation owing to associations with adverse neonatal and neurodevelopmental outcomes. Older adults are more sensitive to sedative, cognitive, and cardiovascular effects and to drug interactions, and patients with psychiatric illness, substance use disorders, or cardiovascular disease face elevated risk [1,77,85].

#### 3.6.4. Drug–Drug Interactions

Cannabinoids interact pharmacokinetically and pharmacodynamically with many co-administered drugs. CBD inhibits CYP2C19, CYP3A4, and several UGT isoforms, while both CBD and THC are substrates of CYP3A4 and CYP2C subfamily enzymes, so inhibitors (e.g., ketoconazole) raise and inducers (e.g., rifampin) lower cannabinoid exposure. The clinically best-characterized interaction is between CBD and clobazam: CBD raises levels of the active metabolite N-desmethylclobazam by more than 200%, potentiating sedation [47]. Co-administration with valproate increases the risk of hepatotoxicity, and additive CNS depression occurs with benzodiazepines, opioids, alcohol, and other sedatives. Cannabinoids may also affect the metabolism of certain antiepileptics and warfarin (via CYP2C9), warranting INR monitoring [33,47,79]. Table 5 summarizes the most clinically relevant cannabinoid drug–drug interactions. Pharmacogenomic variation modulates these interactions and their clinical consequences. CYP2C9 poor metabolizers (e.g., CYP2C9*3 carriers) show higher systemic exposure to THC and its active 11-hydroxy metabolite and appear more susceptible to sedation and other dose-related adverse effects, whereas CYP2C19 loss-of-function alleles raise concentrations of the active 7-hydroxy-CBD metabolite. These observations suggest that genotype-informed starting doses and heightened clinical monitoring may be warranted in known poor metabolizers, although prospective validation in cannabinoid therapeutics is still lacking.

### 3.7. Clinical Practice Guidelines and Recommendations

#### 3.7.1. Professional Society Positions

Professional-society guidance is converging on a cautious, condition-specific stance. The American College of Physicians (2025) conditionally supports a trial of cannabis or cannabinoids for painful neuropathy refractory to first-line therapy while emphasizing small benefits and meaningful harms [57]. ASCO (2024) conditionally recommends dronabinol or nabilone for refractory CINV but not for cancer pain [48], and NCCN lists cannabinoids among breakthrough antiemetic options [51]. The American Psychiatric Association advises against cannabis as a treatment for psychiatric disorders, and addiction-medicine and cardiovascular bodies (including via the AHA statement) caution against use in patients with mental illness or cardiovascular disease [32,77,86]. The VA/DoD recommends against cannabis for PTSD [72]. Across guidelines, the recurring themes are restriction to refractory cases, preference for standardized pharmaceutical-grade products, and avoidance in high-risk populations.

#### 3.7.2. Regulatory Considerations

A persistent tension exists between the small set of FDA-approved cannabinoid medicines—supported by controlled trials and manufactured to consistent specifications—and the large, heterogeneous universe of state-regulated medical cannabis and hemp-derived products that have not undergone comparable evaluation and whose composition is frequently mislabeled. Legal status varies widely across and within jurisdictions, complicating prescribing, insurance coverage, and research. Clinicians should recognize that efficacy and safety data generated with standardized products may not transfer to the dispensary or retail products patients actually obtain [1,6,7]. This regulatory heterogeneity is most acute in the United States, where a federal–state conflict is distinctive: cannabis remains federally Schedule I even as most states operate medical or adult-use programs, and the 2018 Farm Bill created a separate, minimally regulated hemp-derived cannabinoid market. Comparable but not identical fragmentation exists elsewhere—prescription nabiximols and CBD medicines are regulated centrally in the European Union and the United Kingdom, whereas access schemes differ markedly across Canada, Australia, and individual EU member states. We retain this section deliberately: because the efficacy and safety evidence reviewed here is generated almost entirely with pharmaceutical-grade products, the regulatory status—and hence the actual composition—of the product a patient receives is not peripheral but is a determinant of whether that evidence applies at all, and is therefore integral to the clinical interpretation this review is intended to support.

#### 3.7.3. Shared Decision-Making Framework

Given modest benefits, real risks, and product variability, cannabinoid use is best approached through structured shared decision-making. This entails eliciting patient values and treatment goals; conducting an explicit, condition-specific risk–benefit assessment that incorporates age, psychiatric and cardiovascular history, pregnancy status, and concomitant medications; screening for and monitoring cannabis use disorder; preferring non-intoxicating, standardized formulations and the lowest effective dose; and establishing clear stopping rules and follow-up. Harm-reduction counseling—avoiding smoking and driving while impaired and avoiding use in adolescents and during pregnancy—should accompany any decision to proceed [1,57,77].

### 3.8. Knowledge Gaps and Future Directions

#### 3.8.1. Research Priorities

The evidence base remains constrained by short trial durations, small samples, heterogeneous and frequently non-standardized products, and a paucity of head-to-head comparisons with established therapies. Priorities include: adequately powered, long-term (>6-month) randomized trials with standardized formulations; direct comparisons against guideline-recommended treatments; dose-finding and ratio-optimization studies (particularly for THC: CBD combinations); identification of biomarkers and pharmacogenomic predictors of response and adverse effects (e.g., CYP2C9/CYP2C19 polymorphisms); and rigorous surveillance of long-term cognitive, psychiatric, and cardiovascular safety [1,57,87].

#### 3.8.2. Translational Challenges

Bridging the persistent gap between robust preclinical neuroprotection and disappointing clinical results will require attention to dosing equivalence, blood–brain barrier penetration, timing of intervention relative to disease stage, choice of clinically meaningful endpoints, and the standardization of investigational products. Clearer regulatory pathways that allow rigorous evaluation of multi-component botanical extracts—not only single molecules—would accelerate translation [31,37,39].

**Figure 3:**
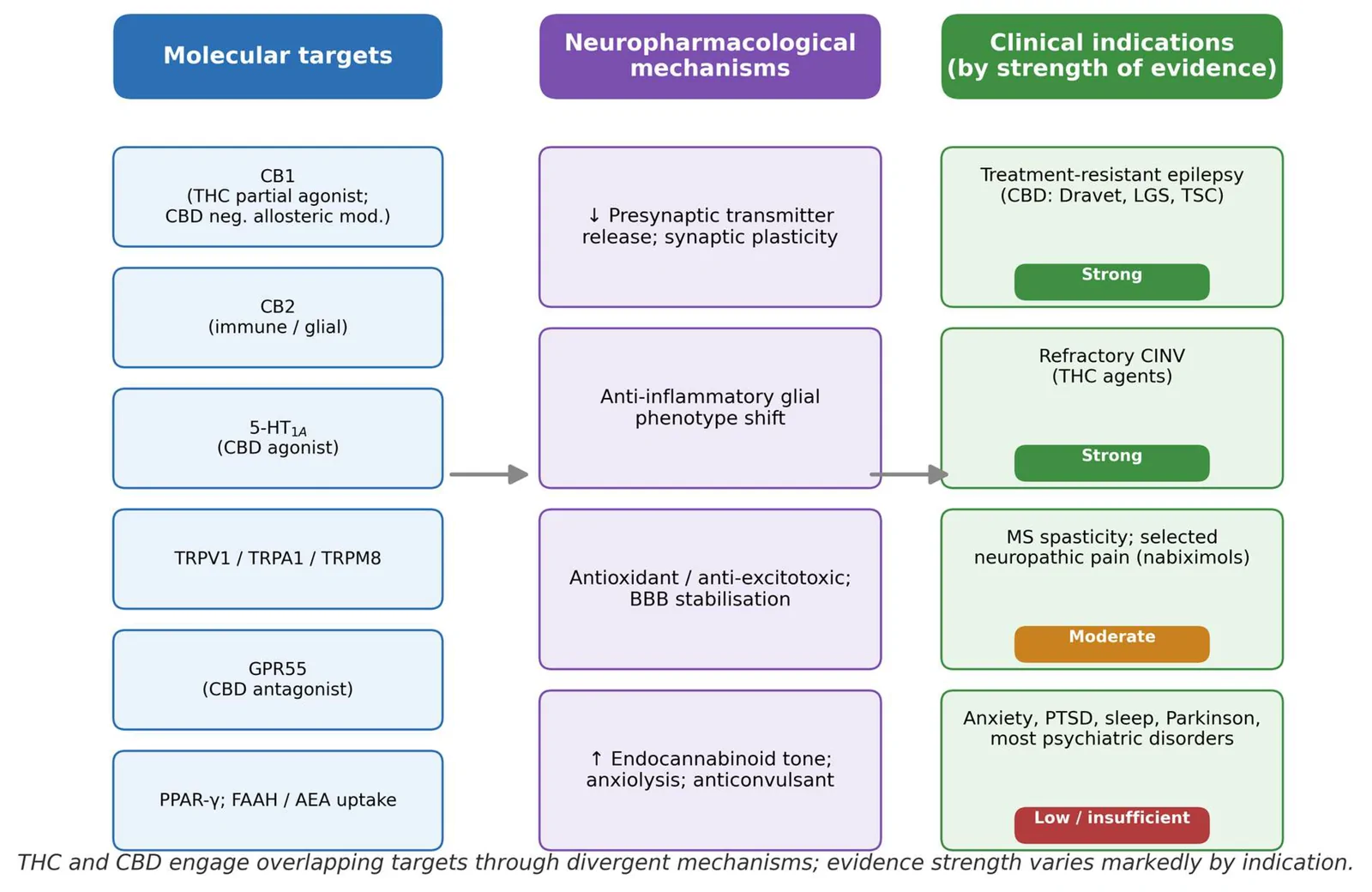
Graphical summary linking molecular targets to neuropharmacological mechanisms to clinical indications graded by strength of evidence. THC and CBD engage overlapping targets through divergent mechanisms; the strength of clinical evidence varies markedly by indication, from strong (treatment-resistant epilepsy, refractory CINV) through moderate (MS spasticity, selected neuropathic pain) to low or insufficient (anxiety, PTSD, sleep, Parkinson’s disease, and most psychiatric disorders). Arrows (→) indicate the progression from molecular targets to neuropharmacological mechanisms to clinical indications. Color coding of the clinical-indication panels denotes the strength of evidence (green, strong; amber, moderate; red, low or insufficient); the column-header colors (blue, purple, green) are organizational only.

Viewed critically, the field is marked by a striking asymmetry between mechanistic and clinical maturity. Preclinical neuroprotection is broad, reproducible, and mechanistically coherent, yet the clinical signal is confined to a few indications and is, for most conditions, either absent or of low certainty once trial size, blinding, sponsorship, and outcome selection are accounted for. The most consistent lesson across conditions is that demonstrable benefit tracks with product standardization and pharmaceutical-grade manufacturing rather than with the breadth of preclinical promise: where standardized single-molecule or fixed-ratio products have been tested in adequately powered RCTs (epilepsy, refractory CINV, MS spasticity), benefit is reproducible, whereas heterogeneous herbal products consistently underperform. Table 6 juxtaposes the preclinical and clinical evidence streams by indication to make this gap explicit, and Figure 3 maps molecular targets through mechanisms to graded clinical indications.

#### 3.8.3. Emerging Areas

Promising frontiers include the minor phytocannabinoids—CBG, CBC, CBDV, and THCV—which show neuroprotective, anticonvulsant, antinociceptive, and antipsychotic-like activity in preclinical models but remain almost entirely untested clinically, with only a single clinical study identified in a recent systematic review of minor cannabinoids for psychiatric disorders [12,14,88]. Other directions include selective and peripherally restricted receptor modulators, enzyme inhibitors that raise endocannabinoid tone (FAAH and MAGL inhibitors), rational cannabinoid–terpene combinations to test the entourage hypothesis prospectively, and personalized-medicine approaches integrating pharmacogenomics and chemovar standardization [17,20,22].

### 3.9. Publication Bias

Publication and reporting biases deserve explicit consideration because they plausibly inflate the apparent efficacy of cannabinoids. In the preclinical literature, positive neuroprotective and antinociceptive results are likely over-represented relative to null findings, and few animal studies are pre-registered or blinded. In the clinical literature, many trials are small, industry-sponsored, and enriched for treatment-resistant populations; several meta-analyses in chronic pain and psychiatric indications have formally detected small-study effects and funnel-plot asymmetry, and selective outcome reporting is common where patient-reported endpoints diverge from objective measures. Because this review is narrative rather than systematic, we did not perform quantitative funnel-plot or Egger testing; readers should therefore interpret the smaller and less certain effect estimates reported here as likely upper bounds, and weight adequately powered, pre-registered, industry-independent trials most heavily.

### 3.10. Strengths and Limitations of This Review

This review has several limitations that should temper its conclusions. First, it is a comprehensive narrative rather than a systematic review: although the literature search was structured and broad, study selection and synthesis were not governed by a pre-registered protocol, a PRISMA flow diagram, formal risk-of-bias scoring, or quantitative meta-analysis, so selection and interpretation bias cannot be excluded. Second, the evidence base is heterogeneous—differing cannabinoid formulations, doses, THC:CBD ratios, routes of administration, comparators, and outcome measures limit direct comparison across studies and the precision of the effect-size estimates we report. Third, much of the preclinical neuroprotection data derives from acute lesion models and may not translate to chronic human neurodegeneration, while many clinical trials are small, short, industry-sponsored, or enriched for treatment-resistant populations, constraining generalizability. Fourth, persistent product variability and inaccurate labeling in state-regulated and consumer markets mean that findings from pharmaceutical-grade agents cannot be extrapolated to most widely available products. Fifth, the rapidly evolving regulatory and commercial landscape, together with our restriction to English-language literature through May 2026, means that some emerging and non-English data were necessarily excluded. Finally, we deliberately excluded synthetic high-potency receptor agonists, so our safety conclusions do not extend to those compounds. These limitations underscore the need for cautious interpretation and for the rigorous, standardized trials called for above.

## 4. Conclusions

Cannabinoids occupy a genuine but circumscribed place in contemporary neurotherapeutics. The neuropharmacology is now well defined: THC and CBD act through divergent mechanisms on a sophisticated endocannabinoid system and a network of non-cannabinoid targets, and these mechanisms rationalize both the therapeutic and adverse effects observed clinically. The strongest evidence supports purified CBD for specific treatment-resistant epilepsies, THC-based antiemetics for refractory CINV, dronabinol for AIDS-related anorexia, and nabiximols for MS spasticity and, to a lesser degree, neuropathic pain. For most other promoted indications—including acute pain, insomnia, and the majority of psychiatric disorders—the evidence is insufficient or negative.

These benefits must be weighed against substantial harms: a high prevalence of cannabis use disorder among medicinal users, neurocognitive and psychiatric risks concentrated in young people and those with psychiatric vulnerability, cardiovascular and respiratory effects, and clinically important drug interactions—compounded by pervasive product variability. The responsible path forward couples cautious, guideline-concordant, shared-decision-making use of standardized products in appropriate patients with a research agenda built on rigorous, long-term, comparative trials. Realizing the considerable therapeutic potential suggested by preclinical science will depend on closing the translational gap with the same methodological rigor applied to any other class of central nervous system medicines.

## Figures and Tables

**Table 3:** Pharmaceutical-grade cannabinoids: approved indications, dosing, and approximate effect sizes.

Product	Cannabinoid	Approved/Best-Evidence Indication	Typical Dose	Effect Size (Certainty)
Epidiolex	CBD (purified)	Dravet, Lennox–Gastaut, tuberous sclerosis complex (FDA)	10–25 mg/kg/day	Seizure reduction SMD ≈ −0.5 (moderate–high)
Dronabinol	Synthetic THC	Refractory CINV; AIDS anorexia (FDA)	2.5 mg BID, up to 10 mg BID	CINV SMD ≈ −0.2 to −0.3; weight SMD ≈ 0.5 (low–very low)
Nabilone	Synthetic THC analogue	Refractory CINV (FDA)	1 mg BID, up to 6 mg/day	Moderate antiemetic; neuropathic pain −1.6 pts (low)
Nabiximols (Sativex)	THC:CBD 1:1	MS spasticity; neuropathic pain (non-US approval)	Titrate to 6–12 sprays/day (≈16–32 mg THC)	Spasticity SMD ≈ −1.4; pain ≈ −0.9 (moderate)

CINV, chemotherapy-induced nausea and vomiting; MS, multiple sclerosis; SMD, standardized mean difference; BID, twice daily. Effect sizes are approximate and pooled across heterogeneous studies; see Refs [1,30,33,48,49,50,55,57,60].

**Table 4:** Representative dosing and titration by indication.

Indication	Agent	Starting Dose	Maintenance/Titration
Treatment-resistant epilepsy	CBD (Epidiolex)	2.5 mg/kg BID	→5 mg/kg BID after 1 wk; max 10 mg/kg BID (12.5 for TSC)
Refractory CINV	Dronabinol/Nabilone	Dronabinol 2.5 mg BID; Nabilone 1 mg BID	Dronabinol to 10 mg BID; Nabilone to 6 mg/day
AIDS anorexia	Dronabinol	2.5 mg BID before meals	Up-titrate as tolerated
MS spasticity/neuropathic pain	Nabiximols	1 spray/day	↑1 spray/day to 6–12/day; reassess at 4 wk (≥20% response)
Chronic noncancer pain	CBD-predominant ± THC	CBD 5 mg BID; add THC 1–2.5 mg	CBD ↑~10 mg q2–3 d to ~40 mg/day; THC ↑1–2.5 mg slowly
Anxiety (investigational)	CBD	150–300 mg/day	Acute anxiolysis ≈ 300–600 mg; inverted-U response

BID, twice daily; TSC, tuberous sclerosis complex; CINV, chemotherapy-induced nausea and vomiting; MS, multiple sclerosis. Compiled from Refs [33,48,52,57,62,66,82,83]. Arrows: →, titrate to/then; ↑, increase.

**Table 5:** Clinically relevant cannabinoid drug–drug interactions.

Mechanism/Enzyme	Affected Drugs	Clinical Consequence and Management
CBD inhibits CYP2C19	Clobazam (→N-desmethylclobazam), diazepam, omeprazole	↑Active metabolite > 200%; increased sedation—monitor and reduce clobazam dose
CBD/THC via CYP3A4	Ketoconazole, clarithromycin (inhibitors); rifampin, carbamazepine (inducers)	Inhibitors ↑ and inducers ↓ cannabinoid levels—adjust dose and monitor response
Hepatic/UGT	Valproate	↑Risk of transaminase elevation—monitor LFTs at baseline, 1, 3, 6 months
Additive CNS depression	Opioids, benzodiazepines, alcohol, sedative-hypnotics	↑Sedation, psychomotor impairment, fall risk—counsel and avoid stacking
CYP2C9 (THC)	Warfarin	Possible ↑INR/bleeding risk—monitor INR closely

LFTs, liver function tests; INR, international normalized ratio. Compiled from Refs [33,47,79]. Arrows: ↑, increase; ↓, decrease.

**Table 6:** Preclinical versus clinical evidence for cannabinoids, by indication.

Indication	Preclinical Evidence	Clinical Evidence (Design and Certainty)	Key References
Treatment-resistant epilepsy	Anticonvulsant via TRPV1/GPR55/adenosine (in vivo)	Multiple pivotal RCTs; FDA-approved (high certainty)	[30,33,43,44,45,46]
Chemotherapy-induced nausea/vomiting	Antiemetic at receptor level (preclinical)	Older RCTs plus recent phase II/III RCTs; guideline-endorsed add-on (moderate)	[48,49,50]
MS spasticity	CB1/CB2 modulation of motor circuits	Meta-analyses of RCTs; moderate certainty (patient-reported)	[60,61]
Neuropathic pain	Robust antinociception across models	Systematic reviews/meta-analyses of RCTs; small benefit, low–moderate certainty	[53,54,55,56]
Parkinson’s disease	Strong dopaminergic neuroprotection	Small, inconsistent trials; insufficient for motor outcomes	[34,36,38,64]
Alzheimer’s/Huntington’s disease	Neuroprotection; ↓ amyloid/tau; ↑ survival	Small trials largely negative on primary outcomes	[37,39,88]
Anxiety disorders	Anxiolytic via 5-HT1A	Small RCTs (e.g., simulated public speaking); insufficient for recommendation	[65,66,68]
Psychotic disorders	CBD opposes THC effects (preclinical)	Early RCT signal; not established (low certainty)	[75,76,77]

RCT, randomized controlled trial; MS, multiple sclerosis. Preclinical and clinical evidence streams are presented separately in response to reviewer request; certainty reflects the highest-quality synthesis available for each indication. Arrows: ↑, increase; ↓, decrease.

## Data Availability

No new data were created or analyzed in this study. Data sharing is not applicable to this article.

## Abbreviations

2-AG, 2-arachidonoylglycerol; AEA, anandamide; CB1/CB2, cannabinoid receptor type 1/2; CBD, cannabidiol; CBDV, cannabidivarin; CBG, cannabigerol; CBC, cannabichromene; CINV, chemotherapy-induced nausea and vomiting; CUD, cannabis use disorder; ECS, endocannabinoid system; FAAH, fatty acid amide hydrolase; MAGL, monoacylglycerol lipase; MS, multiple sclerosis; PPAR-γ, peroxisome proliferator-activated receptor gamma; SMD, standardized mean difference; THC, tetrahydrocannabinol; THCV, tetrahydrocannabivarin; TRP, transient receptor potential.
